# Elevated serum leptin is associated with attenuated reward anticipation in major depressive disorder independent of peripheral C-reactive protein levels

**DOI:** 10.1038/s41598-023-38410-4

**Published:** 2023-07-13

**Authors:** Kaiping Burrows, Breanna A. McNaughton, Leandra K. Figueroa-Hall, Philip A. Spechler, Rayus Kuplicki, Teresa A. Victor, Robin Aupperle, Sahib S. Khalsa, Jonathan B. Savitz, T. Kent Teague, Martin P. Paulus, Jennifer L. Stewart

**Affiliations:** 1 Laureate Institute for Brain Research, 6655 South Yale Ave, Tulsa, OK 74136 USA; 2 Oxley College of Health Sciences, University of Tulsa, Tulsa, OK USA; 3 Departments of Surgery and Psychiatry, School of Community Medicine, The University of Oklahoma, Tulsa, OK USA; 4 Department of Biochemistry and Microbiology, The Oklahoma State University Center for Health Sciences, Tulsa, OK USA; 5 Department of Pharmaceutical Sciences, The University of Oklahoma College of Pharmacy, Oklahoma City, OK USA

**Keywords:** Reward, Molecular neuroscience

## Abstract

Major depressive disorder (MDD) is associated with immunologic and metabolic alterations linked to central processing dysfunctions, including attenuated reward processing. This study investigated the associations between inflammation, metabolic hormones (leptin, insulin, adiponectin), and reward-related brain processing in MDD patients with high (MDD-High) and low (MDD-Low) C-reactive protein (CRP) levels compared to healthy comparison subjects (HC). Participants completed a blood draw and a monetary incentive delay task during functional magnetic resonance imaging. Although groups did not differ in insulin or adiponectin concentrations, both MDD-High (Wilcoxon *p* = 0.004, *d* = 0.65) and MDD-Low (Wilcoxon *p* = 0.046, *d* = 0.53) showed higher leptin concentrations than HC but did not differ from each other. Across MDD participants, higher leptin levels were associated with lower brain activation during reward anticipation in the left insula (*r* = − 0.30, *p* = 0.004) and left dorsolateral putamen (*r* = -− 0.24, *p* = 0.025). In contrast, within HC, higher leptin concentrations were associated with higher activation during reward anticipation in the same regions (insula: *r* = 0.40, *p* = 0.007; putamen: *r* = 0.37, *p* = 0.014). Depression may be characterized by elevated pro-inflammatory signaling via leptin concentrations through alternate inflammatory pathways distinct to CRP.

## Introduction

Major depressive disorder (MDD) affects approximately 163 million people and is the leading cause of years lived with disability worldwide^1^. Only one third of depressed individuals remit with their first antidepressant treatment^2^ and treatment response is known to be limited and difficult to predict^3^. Although anhedonia—inability to feel pleasure—is one of the core features of a major depressive episode and one of the most treatment-resistant symptoms of MDD^4^, little is known about mechanisms implicated in dysfunctional reward processing to inform treatment.

Depressive symptoms are linked to inflammation involving pro-inflammatory cytokines^5–7^ with meta-analyses indicating that interleukin-6 (IL-6), tumor necrosis factor (TNF), and C-reactive protein (CRP) are elevated in MDD patients^8–10^. Approximately half of depressed individuals have a CRP concentration of 3 mg/L or greater, indicating increased risk of future cardiovascular disease^11^. Furthermore, depressed individuals with elevated inflammation show heightened treatment resistance^12,13^ as well as attenuated brain reward processing within the dorsal and ventral striatum^14–17^. Thus, anti-inflammatory drugs may be a potential therapeutic target for a subset of depressed individuals with heightened inflammation^18^.

Complicating the clinical picture, however, is the fact that MDD has a high comorbidity with metabolic syndrome—a cluster of conditions that occur together (e.g., obesity, high blood pressure, cholesterol, hyperglycemia), with an approximate 41% overlap^11^. Although depression, inflammation, and metabolic disorders often co-occur and exacerbate each other^11,19^, mechanisms by which inflammation, metabolism, and impaired reward processing relate to depression remain unclear, thereby diminishing the ability to develop targeted treatment for individuals with MDD presenting with these issues.

Three metabolic hormones—insulin, leptin, and adiponectin—are integral to understanding relationships between inflammation, depression, and brain function. Insulin regulates glucose and energy^20^, while leptin regulates appetite, energy, and mood^21–23^; finally, adiponectin improves insulin sensitivity while increasing food intake and reducing energy^24,25^. Compared to healthy individuals, depressed patients are characterized by: (1) heightened insulin resistance not ameliorated by antidepressants^26^; (2) both increases and decreases in leptin levels^27,28^, with directionality of effects attributable to symptom heterogeneity, age, sex, and medical history^29–31^; and (3) attenuated adiponectin levels^31,32^. While insulin resistance and leptin signaling involve pro-inflammatory processes^33,34^, adiponectin is more closely aligned to anti-inflammatory mechanisms^35–37^. Crucially, all three hormones are thought to be important for dopaminergic reward signaling^38^. In sum, research indicates that insulin, leptin, and adiponectin are linked to inflammation, reward signaling, and depression, but it is still unclear how these hormones relate to altered brain reward processing in MDD as a function of inflammation.

To identify the relationship between metabolic markers, inflammation, and reward processing in MDD, we conducted secondary data analyses, focusing on neural responses to the monetary incentive delay (MID) task and serum metabolic hormone (insulin, leptin, and adiponectin) concentrations in a previously published subset of MDD patients varying in peripheral CRP inflammation^14^. The MID task^39^ reliably recruits the striatum, insula, and thalamus^40^, brain regions that are: (1) implicated in optimal prediction of future rewards^40,41^; (2) sensitized by metabolic hormones^42,43^; and (3) attenuated in depressed individuals^44,45^. We hypothesized that: (a) MDD with high CRP inflammation (MDD-High) would exhibit higher serum leptin and insulin and lower adiponectin concentrations than healthy controls (HC) and MDD patients with normative CRP inflammation (MDD-Low); (b) within MDD-High subjects, higher serum insulin/leptin and lower adiponectin concentrations would be correlated with lower blood oxygen level-dependent (BOLD) signal change within the striatum, insula, and thalamus during anticipation of rewards.

## Results

### Demographics and clinical characteristics

Table 1 shows demographic and clinical characteristics comparing the combined MDD group and HC. There were no differences in age, sex, annual income, education, employment status, nor smoking status between MDD and HC; MDD group showed lower exercise status (*p* < 0.001), higher percent body fat (PBF) (*p* < 0.001), and higher body mass index (BMI) (*p* < 0.001) than HC. Regarding clinical ratings, MDD and HC differed on all PROMIS scores including alcohol use, anger, anxiety, depression, fatigue, physical function, sleep disturbance, and social isolation ratings (*p* ≤ 0.001), except nicotine dependence.

**Table 1:** Sample demographics and clinical characteristics.

	MDD	HC	*p* value
	Mean (SD)	Mean (SD)	
*N*	88	44	
Age (years)	34.34 (11.06)	30.91 (10.15)	0.087^a^
Sex = male (%)	27 (30.7)	19 (43.18)	0.220^b^
Annual income (US dollars)	65,352 (115,668)	51,686 (35,399)	0.447^a^
Consolidated education	6.20 (1.66)	6.50 (1.42)	0.300^a^
Employed = yes (%)	53 (61.6)	32 (78.0)	0.102^b^
Smoke = yes (%)	18 (20.5)	5 (11.4)	0.292^b^
Exercise = yes (%)	32 (36.4)	31 (70.5)	**< 0.001**^**b**^
IPAQ category (%)			**0.002 **^**b**^
HEPA active	28 (35.0)	24 (64.9)	
Inactive	33 (41.2)	4 (10.8)	
Minimally active	19 (23.8)	9 (24.3)	
IPAQ minutes per week	3021.66 (3550.23)	5397.61 (4044.80)	**0.002**^**a**^
Percent body fat	39.18 (8.08)	29.13 (10.84)	**< 0.001**^**a**^
Body mass index (kg/m^2^)	30.56 (4.57)	26.50 (5.22)	**< 0.001**^**a**^
Medication = un-medicated (%)	26 (29.5)	NA	NA
PROMIS alcohol use	48.91 (7.12)	44.52 (6.78)	**0.001**^**a**^
PROMIS anger	58.11 (6.68)	44.38 (5.56)	**< 0.001**^**a**^
PROMIS anxiety	63.08 (6.50)	45.60 (7.42)	**< 0.001**^**a**^
PROMIS depression	61.02 (7.18)	43.50 (6.38)	**< 0.001**^**a**^
PROMIS fatigue	61.20 (7.58)	43.61 (7.14)	**< 0.001**^**a**^
PROMIS nicotine dependence	28.07 (10.84)	25.32 (7.04)	0.129^a^
PROMIS physical function	50.04 (7.05)	60.22 (6.64)	**< 0.001**^**a**^
PROMIS sleep disturbance	57.31 (10.00)	43.67 (7.67)	**< 0.001**^**a**^
PROMIS social isolation	58.15 (5.43)	42.69 (7.89)	**< 0.001**^**a**^
Current major depressive episode on MINI = yes (%)	82 (93.2)	0 (0)	**< 0.001**^**b**^

Significant values are in bold.

IPAQ, International Physical Activity Questionnaire; PROMIS: Patient-Reported Outcomes Measurement Information System total score. MINI, Mini International Neuropsychiatric Inventory; NA = not applicable.

^a^Two sample *t *test.

^b^*χ*^2^ test.

### Immunoassay results

**Figure 1:**
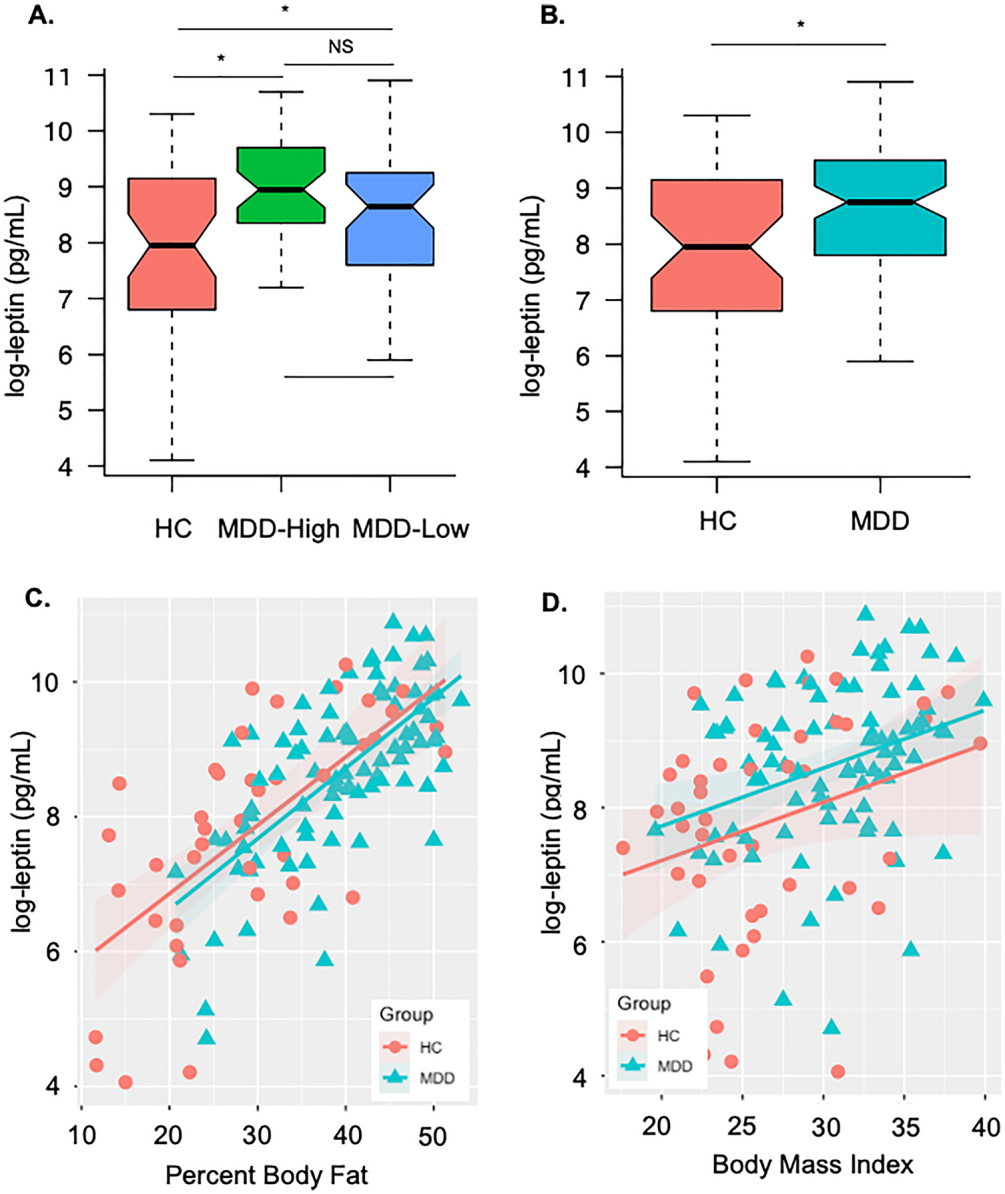
Serum leptin concentrations between groups and their relationship with percent body fat and body mass index. (**A**) Both major depressive disorder (MDD) subjects with high CRP (MDD-High) and low CRP (MDD-Low) exhibited higher log-transformed serum leptin concentrations compared to healthy comparison subjects (HC). (**B**) The combined MDD group showed higher log-transformed serum leptin concentrations than HC. (**C**) Leptin concentrations were positively correlated with percent body fat in MDD and HC groups. (**D**) Leptin concentrations were positively correlated with body mass index in MDD group and partially correlated with BMI in HC group.

Although ANOVA tests indicated that MDD-High, MDD-Low, and HC groups did not differ on insulin or adiponectin concentrations, group differences were observed in serum leptin concentrations (Kruskal–Wallis *χ*^2^ = 8.87, *p* = 0.012). Specifically, both MDD-High (Wilcoxon *p* = 0.004, *d* = 0.65) and MDD-Low (Wilcoxon *p* = 0.046, *d* = 0.53) showed higher leptin concentrations than HC, while there was no difference between MDD-High and MDD-Low (Fig. 1A). Since no leptin differences were found between the two MDD groups, MDD-High and MDD-Low were combined as one MDD group and compared with HC on neuroimaging and leptin concentrations. A Mann–Whitney–Wilcoxon non-parametric test showed that the MDD group exhibited higher leptin levels than HC (*p* = 0.005, *d* = 0.62) (Fig. 1B).

Higher PBF was associated with higher serum leptin concentrations within the MDD group (*r* = 0.68, *p* < 0.001) and the HC group (*r* = 0.67, *p* < 0.001); however, there was no slope difference on PBF and leptin concentration between MDD and HC (*p* = 0.99) (Fig. 1C). Similarly higher BMI was associated with higher serum leptin concentrations in the MDD group (*r* = 0.32, *p* = 0.002) and the HC group (*r* = 0.28, *p* = 0.07); again, there was no slope difference on BMI and leptin concentration between groups (*p* = 0.62) (Fig. 1D). In addition, leptin concentrations were negatively associated with PROMIS physical function scores within the HC group (*r* =  − 0.53, *p* < 0.001), but not the MDD group (*r* =  −  0.09, *p* = 0.39). No other correlations between leptin and PROMIS scores were observed. Moreover, higher PBF was associated with higher serum CRP concentrations within HC group (*r* = 0.58, *p* < 0.001) but not the MDD group (*r* = 0.15, *p* = 0.155), and there was a slope difference on PBF and CRP concentration between HC and MDD (*p* = 0.034); Similarly, higher BMI was associated with higher serum CRP concentrations within HC group (*r* = 0.57, *p* < 0.001) but not within the MDD group (*r* = 0.16, *p* = 0.139), and there was a slope difference on BMI and CRP concentration between HC and MDD (*p* = 0.018).

### Neuroimaging results

**Figure 2:**
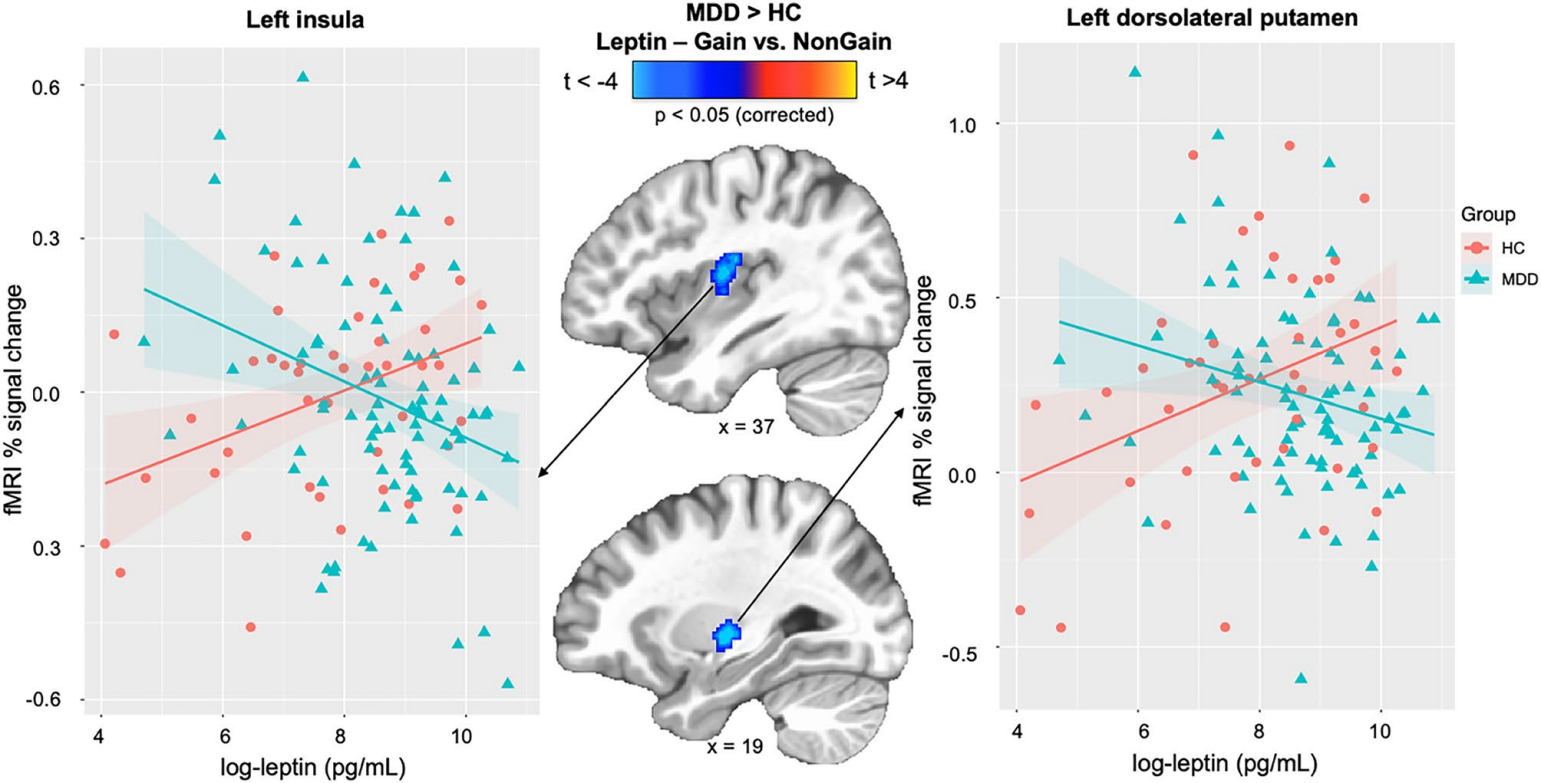
Correlations between serum leptin and % fMRI signal change for the MID gain versus no gain contrast between HC and MDD. Within MDD, higher leptin concentrations were associated with lower fMRI BOLD signal change for the MID gain versus no gain contrast in left insula and left dorsolateral putamen. Within HC, higher leptin concentrations were associated with higher fMRI BOLD signal change for the MID gain versus no gain contrast in left insula and left dorsolateral putamen.

Figure 2 shows that there was a significant slope difference between serum leptin and % fMRI signal change for the MID gain versus no gain contrast between MDD and HC within left mid-posterior insula (center-of-mass = 37.5, 11.3, 13.2; 87 voxels; peak *t* = -3.54) and left dorsolateral putamen (center-of-mass = 28.7, 13.5, − 0.9; 67 voxels; peak *t* = -4.10). Correlations within the MDD patients indicated that those individuals with the highest leptin concentrations also showed the lowest fMRI BOLD signal change for the MID gain versus no gain contrast in left insula (*r* = -  − 0.30, *p* = 0.004) and left dorsolateral putamen (*r* = -  − 0.24, *p* = 0.025). In contrast, within HC, those individuals with the highest leptin concentrations also showed the highest fMRI BOLD signal change for the MID gain versus no gain contrast in left insula (*r* = 0.40, *p* = 0.007) and left dorsolateral putamen (*r* = 0.37, *p* = 0.014). Fisher's *r-to-z* transformations were applied to these correlations for each group and then compared; results indicated that the relationship between serum leptin and gain versus non-gain BOLD signal change was significantly more negative in MDD than HC, within the left insula (*z* =  − 3.88, *p* < 0.001) and the left dorsolateral putamen (*z* =  − 3.31, *p* < 0.001).

In addition, partial correlations showed a significant negative relationship between leptin and gain versus non-gain BOLD signal change within the left insula (*r* =  − 0.29, *p* = 0.006) and the left dorsolateral putamen (*r* =  − 0.23, *p* = 0.033) in the MDD group after controlling for BMI. Similarly, partial correlations within HC subjects showed that the positive relationship between leptin and gain versus non-gain BOLD signal change within the left insula (*r* = 0.37, *p* = 0.014) and the left dorsolateral putamen (*r* = 0.38, *p* = 0.013) remained after controlling for BMI. Moreover, when CRP concentrations were used as a covariate, the significant negative correlation between leptin and gain versus non-gain BOLD signal change within the left insula (*r* =  − 0.30, *p* = 0.005) and the left dorsolateral putamen (*r* =  − 0.24, *p* = 0.025) remained the same within the MDD group, the positive relationship between leptin and gain versus non-gain BOLD signal change within the left insula (*r* = 0.38, *p* = 0.013) and the left dorsolateral putamen (*r* = 0.38, *p* = 0.011) in the HC group remained similar as well.

## Discussion

This study investigated whether two MDD groups varying in inflammation (low versus high CRP levels) and a HC group differed on three metabolic factors—insulin, leptin, and adiponectin—and if so, how variation in metabolism related to brain activation as a function of group while individuals were anticipating future reward. Contrary to our hypotheses, MDD groups with low versus high CRP levels (indicative of differences in peripheral inflammation levels) did not differ from each other on any of the three metabolic hormones, suggesting that peripheral inflammation does not influence metabolic-mediated outcomes in MDD. The combined MDD group also did not differ from the HC group on adiponectin and insulin levels. These findings align with prior work showing no significant relationship between adiponectin and depression^46,47^. Due to adiponectin’s role in both pro- and anti-inflammatory processing in depression, perhaps these competing effects cancel each other out^46^. Study results on insulin concentrations within MDD in other samples are also mixed^26^, suggesting that dysregulated inflammatory and metabolic profiles may not always co-occur in the same subtype of depression. As variation in metabolic profiles may represent different subtypes of depression (e.g., MDD with increased versus decreased appetite, weight change, or atypical vs typical depression)^26,48,49^, perhaps inflammatory and metabolic profiles of depression do not overlap.

Crucially, we demonstrate two important findings for the entire MDD group that are consistent with predictions for the MDD high inflammation group. First, MDD, regardless of inflammation status (as indicated by CRP level), showed greater serum leptin concentrations than healthy individuals, consistent with previous research^27,28^. The absence of leptin-mediated inflammatory function as measured by peripheral CRP concentrations suggests that alternate inflammatory pathways may be involved leptin’s role in reward processing that have yet to be explored^50^. Leptin binding to its receptor leads to canonical signaling via Janus kinases (JAK) and signal transducer and activator of transcription protein (STAT) pathways^51^, but also has the capability of extracellular signal-regulated kinases (ERK), p38 mitogen-activated protein kinases (MAPK), c-Jun N-terminal kinases (JNK), protein kinase C (PKC), and phosphatidylinositol 3-kinase (PI3K) activation^50^. These pathways, all of which have been implicated in depression, mediate leptin signaling on targets affecting neurotransmitter regulation, antidepressant activity, neuronal spine density, and glucocorticoid resistance^52–56^.

Second, left mid-posterior insula and left dorsolateral putamen responses during anticipation of reward were lowest in MDD patients with the highest leptin concentrations, but this pattern was reversed in healthy individuals (higher left insula and left putamen responses were linked to higher leptin levels). Taken together, these findings suggest that depression may be characterized by elevated pro-inflammatory signaling via leptin concentrations through alternate inflammatory pathways distinct to CRP, which relates to blunted mid-insula signals previously evident in MDD patients during the processing of bodily sensations^57,58^ as well as reduced insula and putamen responses during reward anticipation in girls at high risk for MDD^59^. As reduced striatal signal during reward processing may precede MDD onset^60^, it will be important for future research to determine whether high leptin levels are also present in individuals at high risk for future MDD. We speculate that perhaps elevated leptin and attenuated striatum activity might interact during adolescence to reduce positive mood and appetitive valuation of reward stimuli. Leptin regulates brain reward and motivation signals in striatal regions, including the caudate^61^; however elevated leptin levels may inhibit dopamine release and therefore reduce reward activation in the caudate, which may be associated with a high fat diet^62^. Prior work in our research group found that MDD patients had poor diet quality, including a high fat diet^63^. A healthy dietary pattern would be useful to reduce leptin concentrations, which may then increase reward activity in striatal regions^64^. Although leptin receptors were observed in the insular cortex in animal studies^65^, it is unclear whether neurons that contain leptin receptors can sense the peripheral leptin to regulate reward processing. Leptin signaling within the insular cortex may alter brain function^66^ through both JAK2-MAPK and JAK2-PI3K pathways, conceivably via elevated leptin concentrations (leptin resistance) leading to decreased reward processing in depressed individuals. Another pathway to reward processing could be the sympathetic activation that is modulated by leptin. It is possible that individuals with or at high risk for MDD lack these leptin-sensitive neurons^67^.

In our study, MDD differed in exercise, percent body fat (PBF), body mass index (BMI), and PROMIS scores compared to HC. MDD participants had lower exercise scores, as measured by the International Physical Activity Questionnaire, which may be attributed to depressive symptoms, higher BMI, and physical co-morbidity with other illnesses^68^. Despite the evidence that exercise/physical activity improves mental health parameters^69,70^, not all individuals with depression engage in physical activity or to levels that would be beneficial^71,72^. Also, MDD subjects showed higher PBF and BMI than HC and were both positively associated with leptin as previously reported^73,74^. Higher PBF and BMI are associated with increased risk for obesity, which is often comorbid with MDD and associated with a distinct set of depressive symptoms^75–78^. In our study, PBF and BMI are also highly correlated with CRP in HC group, but not in MDD group, which may explain elevated leptin concentrations regardless of CRP-mediated inflammatory status in MDD^79^. Leptin, which is synthesized in adipose tissue and influences body weight, may be linked to this bidirectional relationship between obesity and depression, due to higher levels of adipose in depressed individuals^75,80^. Lastly, PROMIS scores, except nicotine dependence, also differed in MDD versus HC. It is not surprising that PROMIS Anger, Anxiety, and Depression were all higher in MDD versus HC, which represents more negative affect^81^. While PROMIS Physical Function was different in MDD versus HC, it was only associated with leptin in HC (not MDD) suggesting that leptin may not affect physical function in depressed individuals.

This project possesses several strengths, including: (1) the integration of blood-based inflammation and metabolic data with functional neuroimaging; (2) a sizable sample of MDD patients (*n* = 88); and (3) analysis of the MID paradigm that enables separation of reward anticipation and outcome phases. However, this project focused on secondary analysis of the Tulsa 1000 project data, which also includes multiple limitations. First, most of the insulin research in depression has specifically focused on insulin resistance as opposed to total insulin concentration as measured in the Tulsa 1000 project. Second, in contrast to prior work selecting MDD patients on particular characteristics relevant to appetite, weight, or atypical symptoms when investigating metabolic factors linked to depression^26,48,49^, individuals with MDD from the Tulsa 1000 project were not selected on the basis of these criteria. Third, as research indicates that body fat and leptin are positively correlated^30^, it is imperative to note that MDD patients in our sample had a higher BMI than HC that also positively scaled with leptin levels; however, within our MDD group, BMI was not significantly related with insula and putamen activation during reward anticipation and did not account for the negative correlation between leptin and reward-related brain activation. Fourth, although two-thirds of the MDD group were currently taking at least one type of psychotropic medication, medicated and unmedicated MDD participants did not differ on leptin levels, BMI, insula and striatum BOLD signal, or relationships between these metrics suggesting that medication was not accounting for the present results.

We show evidence that the association between leptin and anticipatory reward processing is different in depressed versus non-depressed individuals, which may be due to a metabolic re-regulation involving leptin-related pathways. More importantly, our findings suggest that leptin’s mechanism of action to reward processing is not dependent on inflammation as related to CRP peripheral concentrations, this may provide us with a new therapeutic window (e.g., increase leptin sensitivity to reward processing) for treatment of the most treatment-resistant depressive symptom—anhedonia.

## Methods

### Participants

A subset of participants varying on peripheral inflammation, as determined by serum CRP levels, was drawn from the first 500 subjects as part of the Tulsa 1000 study, a naturalistic longitudinal study of 1000 individuals with mental illness and HC^82^. The Tulsa 1000 study was approved by the Western Institutional Review Board and conducted in accordance with the Declaration of Helsinki; all participants provided written informed consent and received compensation for their participation. See Victor et al. for the complete Tulsa 1000 study protocol^82^.

See Burrows et al. for the study population used in the present analysis^14^. Briefly, three, age and sex matched groups of subjects were selected based on their serum CRP concentrations: (a) MDD subjects with CRP > 3 mg/L (MDD-High, *n* = 44, CRP range 3.12–22.99 mg/L); (b) MDD subjects with CRP concentrations between 0 and 3 mg/L (MDD-Low, *n* = 44, CRP range 0.07–2.89 mg/L); and (c) HC subjects regardless of their CRP concentrations (HC, *n* = 44, CRP range 0.05–9.47 mg/L). All MDD participants completed the Mini International Neuropsychiatric Inventory (MINI)^83^ and met either Diagnostic and Statistical Manual of Mental Disorders (DSM)–IV or DSM-5 criteria for a major depressive disorder. The three groups were matched on demographics including age, sex, income, education, employment status, and smoking status.

All participants provided blood samples and completed two runs of the functional magnetic resonance imaging (fMRI) scan with the MID task. Venous blood samples, collected in BD Vacutainer serum collection tubes, were centrifuged at 1300×*g* for 10 min at room temperature. The serum was then aliquoted and stored at − 80 °C until analysis.

### Immunoassays

Serum leptin and insulin concentrations were both analyzed using the Human Leptin, Insulin Kit (Meso Scale Diagnostics, Maryland, USA). Adiponectin was measured with the Human Quantikine ELISA kit (R&D Systems, Minneapolis, USA). The Neuroinflammation Panel 1 Human Kit (Meso Scale Diagnostics, Maryland, USA) was used to measure CRP concentrations. All analytes were tested in duplicate. The intra- and inter-assay coefficients of variation (CV) were 6.7% and 8.9% (leptin), 6.9% and 8.5% (insulin), 2.8% and 6.9% (adiponectin), 2.2% and 10.0% (CRP), respectively.

### fMRI MID task

Brain reward processing was measured using the MID task^84^ programmed in PsychoPy^85^. Each MID run included 45 trials and lasted 562 s. On each trial, a cue that indicated a potential win or loss (circle or square) was presented, then a target (white triangle) was presented after a short delay. Participants were instructed to press a button within a short response time to win or avoid losing the amount of money indicated by the cue. To make sure each participant succeeded on approximately 66% of trails, task difficulty was calibrated by each participant’s reaction time during a practice session and updated during the scan. Brain images were acquired with two identical GE MR750 3T scanners at Laureate institute for Brain Research, Tulsa, Oklahoma, USA. The scanning parameters were TR/TE = 2000/27 ms, FOV/slice = 240/2.9 mm, 128 × 128 matrix, 39 axial slices. High resolution structural T1-weighted images were acquired (TR/TE = 5/2.012 ms, FOV/slice = 240 × 192/0.9 mm, 186 axial slices).

### Neuroimaging data preprocessing

Neuroimaging data preprocessing was conducted using the AFNI software package^86^. The preprocessing steps included discarding of first 3 TRs, despiking, slice timing correction, co-registration to anatomical volumes, motion correction, smoothing with a 4 mm Gaussian full width at half-max smoothing kernel, and normalization to Montreal Neurological Institute space. The blood oxygen level dependent (BOLD) response to each of the six anticipatory task conditions (three win and three loss) was modeled with four-second block regressors convolved with a canonical hemodynamic response function. Volumes with either a Euclidean norm of the derivatives of the six motion parameters greater than 0.3 or greater than 10% outlier voxels were removed from regression step. Regressors used in the model were the first 4 polynomial baseline terms, along with 6 motion parameters (roll/pitch/yaw/x/y/z translation), large loss (− 5), small loss (− 1), no loss (− 0), no win (+ 0), small win (+ 1), large win (+ 5).

### Statistical analysis on immunoassays

Normality of leptin, insulin and adiponectin distributions were tested using Shapiro-Wilks test; all three analytes were log-transformed due to their non-Gaussian distributions. Group differences on insulin and adiponectin concentrations were assessed using Analysis of Variance (ANOVA). The distributions for leptin were found to be non-Gaussian even after log-transformation, therefore, Kruskal–Wallis and Mann–Whitney–Wilcoxon non-parametric tests were used to test group differences. Values with an absolute *z* greater than 3 for each analyte were defined as outliers, however, no outliers were found in this dataset. Effect size was computed with Cohen’s d. In addition, Pearson’s correlations were used to explore potential relationships between leptin and demographics/clinical characteristics including: PBF, BMI, exercise, and PROMIS ratings within the MDD or groups. ANOVA tests were used to evaluate leptin level slope differences between groups.

### Statistical analysis on neuroimaging

The slope difference in the relationship between leptin and percent fMRI BOLD signal change on the MID gain versus non-gain contrast was evaluated using AFNI’s group analysis program 3dttest++ using the model beta ~ Group*log(leptin). Clusters with a significant interaction were selected based on a voxel wise p < 0.05. The family wise error rate was set to α < 0.05 using 3dClustsim to estimate probability of false positives and 3dFWHMx to measure the intrinsic smoothness of the residuals, both using the spatial autocorrelation function (acf) option. Small volume correction was performed by applying this cluster-wise correction separately for 10 different regions (left and right insula, thalamus, caudate, putamen, nucleus accumbens) that were selected a priori. Follow up regression analyses were conducted in R for significant clusters.

## Data Availability

The data that support the findings of this study are available on request from the corresponding author, KB. The data are not publicly available due to restrictions e.g., their containing information that could compromise the privacy of research participants.
